# Supplementary figures and images for: Fast and accessible morphology-free functional fluorescence imaging analysis
Source: PLoS Comput Biol. 2026 Mar 12;22(3):e1014038. doi: 10.1371/journal.pcbi.1014038 (PMC13038116; doi:10.1371/journal.pcbi.1014038)

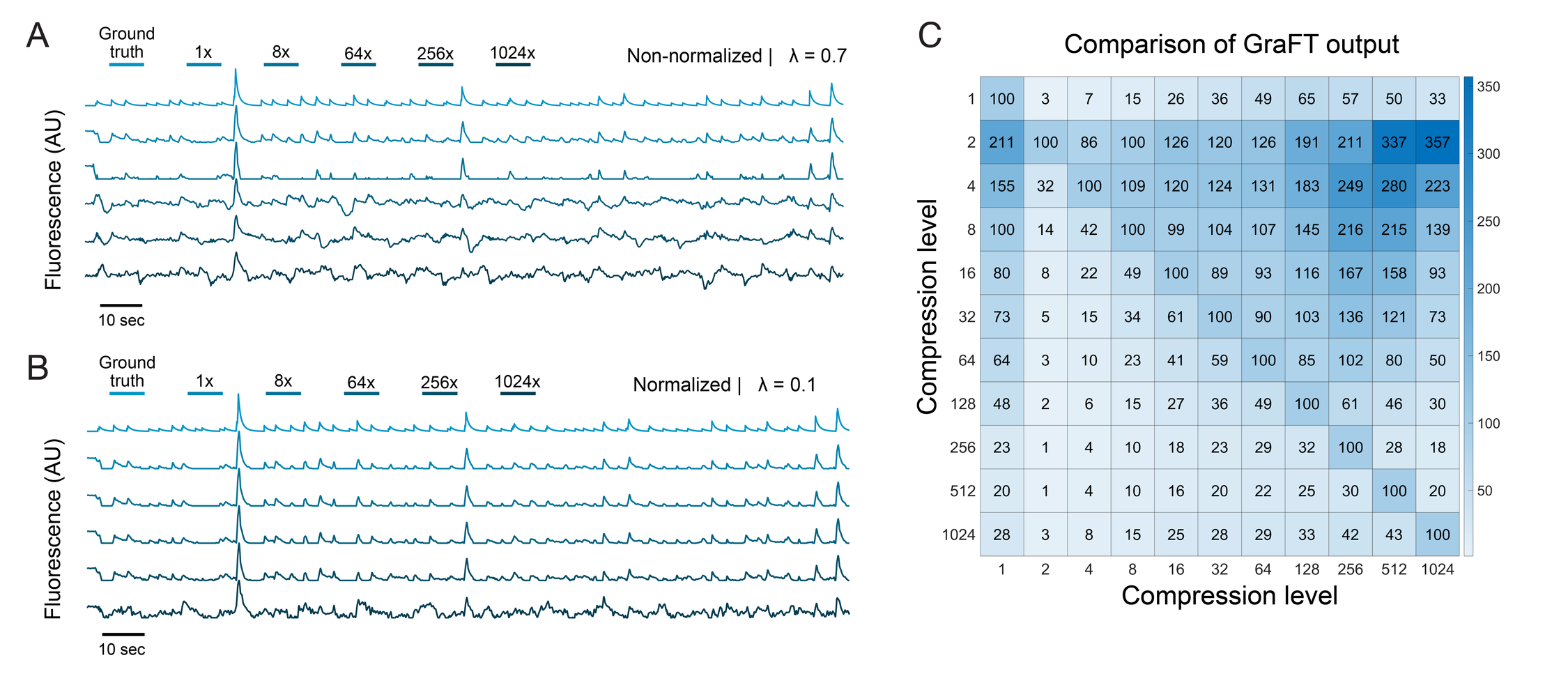

Supplement: S1 Fig — Example medium correlated time traces (0.4<ρ<0.7) across compression levels in (A) non-normalized GraFT and (B) normalized GraFT. (C) Heatmap for normalized GraFT results across compression levels. Values in the heatmap are the actual number of identified traces. (TIF) [file pcbi.1014038.s001.tif]
